# Visual-reward driven changes of movement during action execution

**DOI:** 10.1038/s41598-020-72220-2

**Published:** 2020-09-23

**Authors:** Angela Marti-Marca, Gustavo Deco, Ignasi Cos

**Affiliations:** 1 Center for Brain and Cognition (CBC), Department of Information Technologies and Communications (DTIC), Pompeu Fabra University, Edifici Mercè Rodoreda, Carrer Trias i Fargas 25-27, 08005 Barcelona, Catalonia Spain; 2 Institució Catalana de Recerca I Estudis Avançats (ICREA), Passeig Lluis Companys 23, 08010 Barcelona, Catalonia Spain; 3 Faculty of Mathematics and Informatics, University of Barcelona, Gran Via de les Corts Catalanes 585, 08007 Barcelona, Catalonia Spain; 4 Serra-Húnter Research Programme, 08010 Barcelona, Catalonia Spain; 5 Headache and Neurological Pain Research Group, Department of Medicine, Vall d’Hebron Research Institute, Universitat Autònoma de Barcelona, Barcelona, Spain

**Keywords:** Decision, Motor control, Decision

## Abstract

Motor decision-making is often described as a sequential process, beginning with the assessment of available options and leading to the execution of a selected movement. While this view is likely to be accurate for decisions requiring significant deliberation, it would seem unfit for choices between movements in dynamic environments. In this study, we examined whether and how non-selected motor options may be considered post-movement onset. We hypothesized that a change in reward at any point in time implies a dynamic reassessment of options, even after an initial decision has been made. To test this, we performed a decision-making task in which human participants were instructed to execute a reaching movement from an origin to a rectangular target to attain a reward. Reward depended on arrival precision and on the specific distribution of reward presented along the target. On a third of trials, we changed the initial reward distribution post-movement onset. Our results indicated that participants frequently change their initially selected movements when a change is associated with an increase in reward. This process occurs quicker than overall, average reaction times. Finally, changes in movement are not only dependent on reward but also on the current state of the motor apparatus.

## Introduction

Neural recordings have revealed that pre-motor cortical areas may simultaneously represent several movements options during the delay period of decision-making tasks^1–3^ and that decisions between actions lead to neural competition^4,5^. However, the specifics of how non-selected actions may continue to be represented across the fronto-parietal loop after movement initiation and how they interact with the ongoing motor plan remain unclear. Evidence shows that poor sensory evidence facilitates the change of movement (CoM) post-onset^6^ and that CoMs may be triggered by sudden target jumps or perturbations to the motor apparatus^7^. These events may cause a large enough change of state of the motor apparatus to reverse the initial appeal of possible options and subsequently the final movement choice. Furthermore, experimentally recorded RTs typically last ~ 200–300 ms^8,9^, while CoMs post movement-onset have been quantified as being as short as ~ 150 ms^10,11^. One explanation for this reduction in RTs is that both options had already been assessed and their motor plans prepared prior to movement onset; thus reducing the necessary time to switch to an alternate option after the first movement is ongoing^9,12^. However, while previous studies have examined CoMs between movements caused by sudden changes to the motor apparatus, including physical perturbations^7^, here we wondered how voluntarily enacted CoMs occur in the context of decisions between reward-driven movements post-movement onset. In other words, we focused on CoMs as a response to change in the visual reward associated with each movement. Decisions between movements have been shown to be a trade-off between rewards^13–15^ and costs^16–18^ and we questioned whether this principle holds post-decision and post-movement onset. Our hypothesis is that, in addition to an increase in prospective reward, both the state of the motor apparatus and the available time significantly influence the likelihood to change an initial, ongoing movement. To test this, we performed a decision-making task in which human participants selected and executed a reaching movement, from an origin cue to a wide rectangular target, which was associated to a specific reward distribution. We made reward contingent upon arrival precision, zero in the center of the rectangle, maximum at the right and/or left rectangle vertices and increasing linearly in between. To test our hypothesis, we changed the original distribution at different times after movement onset on approximately one third of trials. Our results show that participants mainly altered their initially selected movement when, according to the new distribution, a better prospect was offered along a path different to their original choice. Furthermore, changes of movement were more frequent for slow movements and occurred on average quicker than the overall reaction time. In summary, this supports the theory that the system simultaneously considers several motor actions post decision-making and strongly suggests the existence of an interaction between motor and reward systems.

## Results

**Figure 1:**
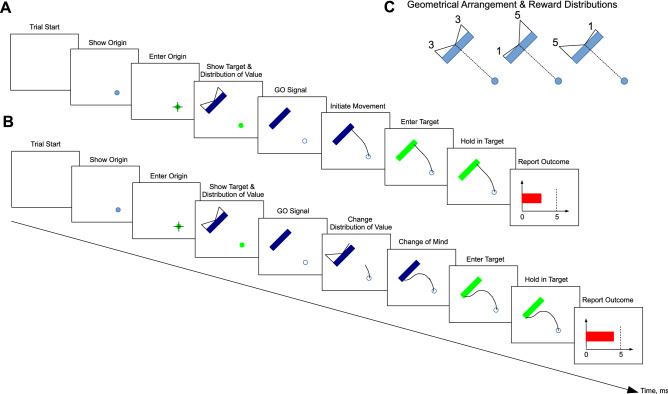
(**A**,**B**) Both baseline and change of movement trials started with the presentation of a blank screen during 500 ms. Next, a circular, pale blue origin cue (1 cm diameter circle) was presented on the bottom right of the screen. One cycle (~ 10 ms) after the end-point entered the origin cue, the cue changed to green and the rectangular target (10 cm long, 1 cm wide) was presented on the top left of the screen, 15 cm away from the center of the origin cue and rotated 135°. Simultaneously, the distribution of visual reward was presented in the form of two right-angled triangles, centered in the middle of the length of the rectangle and peaking on either side. One of three possible distributions was presented: 1–5, 3–3 or 5–1. The GO signal was given 100 ms after the presentation of the distribution, by turning the origin cue white (the background color). On CoM trials, some time post-movement onset, a second distribution of visual reward was presented for a 250 ms duration. Upon arrival at the target, the rectangle color went from blue to green to signal correct entry. A red, horizontal bar provided visual feedback related to arrival precision and its length was proportional to greater reward (and therefore increased precision). (**C**) Geometrical arrangements, associated to the distributions of visual reward, used in this experiment: each DoVR always appeared on top of the rectangular target. One of three possible distributions was presented: 3–3, 1–5, 5–1, shown from left to right respectively.

To test our hypothesis, 15 participants performed a decision-making task. The aim of the task was to maximize visual reward by making planar and highly precise reaching movements from an origin cue to a position along the length of a rectangular target (Fig. 1C). We explained to each participant that the amount of reward obtained would depend on their choice and reaching precision as well as the distribution of visual reward (DoVR) presented on each trial. The DoVR was briefly shown at trial start and could be one of three bimodal distributions: 3–3 (even reward on each side), 1–5 (more reward on the right), 5–1 (more reward on the left)—Fig. 1C. There were two possible trial types: baseline (2/3 of all trials, Fig. 1A) or change of movement trials (CoM; 1/3 of all trials, Fig. 1B), which were randomly interspersed. The only difference between the two types was that a second DoVR, replacing the initial one, was shown sometime after movement onset on CoM trials (see in “Methods” section).

### Baseline movements

**Figure 2:**
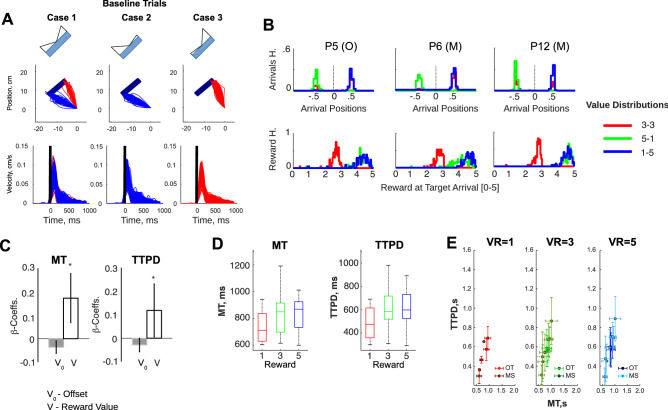
Baseline trial analyses. (**A**) Top: Trajectories for a typical participant (P3, recorded using the Optitrak), plotted as a function of each DoVR: 3–3, 5–1, and 1–5, and color-coded in accordance to their selected target side (right: red; blue: left). Bottom: baseline tangential velocities for each distribution of reward: 3–3, 5–1, and 1–5. (**B**) Distributions of arrival positions and associated reward (top/bottom) for three typical subjects (P6,12: recorded with Computer Mouse—M; P5: recorded using the Optitrak—O), color-coded as described in (**A**). (**C**) Group average β-coefficients for the GLMs of Movement Time (MT) and Time-to-Peak-Deceleration (TTPD) as a function of Visual Reward (V). (**D**) Boxplots of the group MT and TTPD as a function of Visual Reward (V). (**E**) Single participant scatter plots of TTPD vs. MT, with their standard errors, for each Visual Reward value (1, 3, 5).

A set of typical trajectories for baseline trials is shown in Fig. 2A (Participant #2). In this figure, we show trajectories from the origin cue to a position along the wide side of the rectangular target, for all three baseline DoVR shown in Fig. 1C (3–3; 1–5; 5–1). Consistent with instruction, the trajectories favor the side of the rectangle that offers the largest reward in the 5–1 and 1–5 distributions, and evenly favor both sides in the case of the 3–3 distribution. These observations are also reinforced by the distribution of arrival positions and related rewards shown in Fig. 2B for three participants (P5, P6, and P12).

Tangential velocity profiles are shown in Fig. 2A for all three baseline cases, aligned on movement onset. The profiles exhibit a fast rise to peak and a longer deceleration phase until target arrival, consistent with the need for a slower, more controlled movement, during the interval immediately preceding target arrival (and subsequent reward delivery). This notion is reinforced by a positive effect of value on the overall movement time (MT; F(15,1) = 6.95; *p* = 0.018) and on deceleration time (TTPD; F(15,1) = 5.04; *p* = 0.043)—group average regression coefficients are shown in Fig. 2C. This suggests the dynamics of a speed-accuracy trade-off, in which the subject’s primary drive for reward is counterbalanced by the concern for accurate aiming^15,19,20^. Also, Fig. 2E shows scatter plots of the mean and standard error of the TTPD and MT for each individual subject, color-coded as a function of the amount of reward aimed for, and the tracking apparatus (see “Methods” section).

### Change of movement

**Figure 3:**
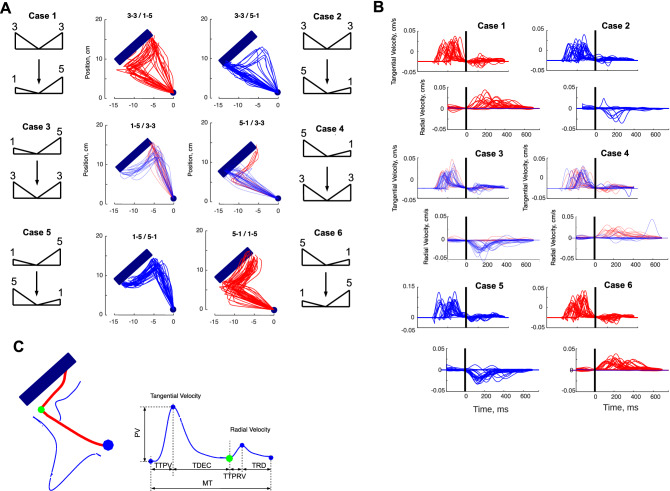
(**A**) Typical Change of Movement (CoM) trajectories for the six types of CoM trials (1. 3–3/1–5; 2. 3–3/5–1; 3. 1–5/3–3; 4. 5–1/3–3; 5. 1–5/5–1; 6. 5–1/1–5) (P9, Computer Mouse). (**B**) CoM tangential and radial velocities for P9, for all six types of CoM trials, aligned at CoM. (**C**) Definition of Kinematic Markers for the tangential velocity (Peak Velocity—PV, Time-To-Peak Velocity—TTPV, Time-to-Deceleration—TDEC), and radial velocity (Time-to-Peak Radial Velocity—TTPRV, Time-to-Radial Deceleration—TRD) of a CoM trial, and overall Movement Time—MT.

Figure 3A shows a set of trajectories during change of movement (CoM) trials, as a function of their initial/final distributions of reward (3–3/1–5; 3–3/5–1; 1–5/3–3; 5–1/3–3; 1–5/5–1; 5–1/1–5). The trajectories confirm that subjects followed the instructions and that their goal was to gain reward, given that target arrival position was most frequently close to the largest reward. However, we identified two major behavioral strategies to attain the desired arrival position. First, similar to baseline trials, the most popular strategy (14/15 participants) consisted of an initial reaching movement towards the side associated with the largest reward, and then altering that ongoing movement if, after the appearance of the second DoVR, the other side now offered a better reward. The second strategy (1/15 participants), for DoVRs where there was a strong imbalance (e.g., 1–5/5–1), consisted of initiating a trajectory towards the least appealing side and changing the motor path only if the second DoVR confirmed the lesser reward. In a way, the first strategy assumes that the initial distribution will not change (there will be no second DoVR), while the second one hopes for a change as the movement progresses. Figure 3B shows a set of typical tangential and radial velocities for all six cases of CoM trials. The velocities are aligned at the time of CoM (vertical black line), which effectively partitions the movement into two distinct phases. First, a ballistic phase, tangential from the origin cue, and second, a radial phase towards the opposite target side.

### Changes of trajectory during ongoing movements

We hypothesized that participants would adjust their reaching movement whenever an alternative option offered some gain in reward with respect to the original choice. Consistent with this, an observation of the trajectories (Fig. 3A) indicated that participants most frequently changed their movement when the second DoVR revealed a better prospect at the other side of the target. Furthermore, to test the potential influence of time and velocity on CoMs, we classified trials as Early/Late and Slow/Fast by performing median splits on the distribution of presentation times of the second DoVR and on the distribution of Peak Velocities preceding the CoM, respectively. Next, we fitted a binomial distribution to the proportion of CoMs experimentally observed for each of the 6 Gain × 2 Times × 2 Velocities cases at a single participant level, obtaining p-parameter values for different analyses of Gains, Times and Velocities (see “Methods” section).

**Figure 4:**
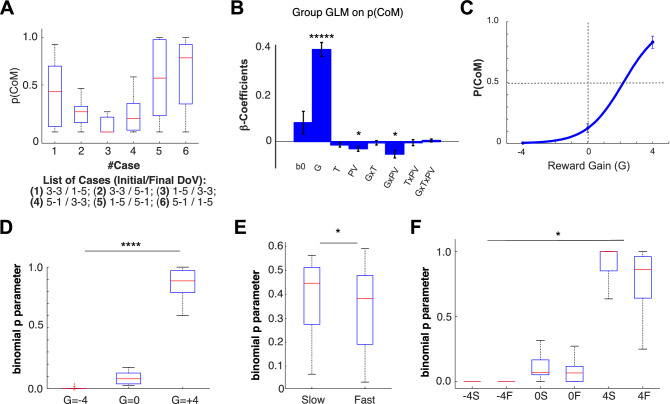
(**A**) Group distribution of the Probability of a Change of Movement (PCoM) for the six types of CoM trials (1. 3–3/1–5; 2. 3–3/5–1; 3. 1–5/3–3; 4. 5–1/3–3; 5. 1–5/5–1; 6. 5–1/1–5). (**B**) Group average and standard error of β-regression coefficients of full GLMs performed on the binomial p parameter fitted to the PCoM, as a function of three factors: reward Gain (G), Time of presentation of the second distribution of visual reward (T), Peak tangential Velocity (PV), and their covariates. The PCoM exhibits a main increasing effect of G (F(15,1) = 120.32, *p* = 1.37E−8) and a decreasing effect with PV (F(15,1) = 6.91, *p* = 0.021), as well as an interaction (F(15,1) = 9.21, *p* = 0.0084) (*****p < 1E−5, ****p < 1E−4, ***p < 1E−3, **p < 0.01, *p < 0.05). (**C**) Group PCoM boxplots as a function of G, fitted using a sigmoid (METHODS). (**D**) Group pCoM (binomial fit p-parameter) boxplots as a function of G. (**E**) Group PCoM boxplots as a function of PV. (**F**) Group PCoM boxplots as a function of G and PV.

To analyze the dependence of the Probability of a Change of Movement (PCoM) on the experimental conditions, we first calculated the binomial p-parameter for all six CoM trial cases and participants (see distribution on Fig. 4A). We also obtained this parameter for each combination of the three experimental factors related to CoM trials: reward Gain (G) associated with the CoM, the time (Early/Late) of presentation of the second DoVR, and the peak velocity (Slow/Fast) prior to the tCoM. Furthermore, to assess statistical dependence, we performed a full GLM of the p-parameter as a function of each of the factors and their covariates within-subjects, including a binary variable for group that signaled the movement tracking method (Computer Mouse/Optitrack) for each subject (see “Methods” section).

The grand average regression coefficients across participants are shown in Fig. 4B. Results from the F-tests show two main group effects on PCoM: a strong positive effect of Gain (F(1,15) = 120.32, *p* = 1.37E−8*), and a negative effect of Peak Velocity (F(1,15) = 6.91, *p* = 0.021*) as well as an additional interaction between the two (F(1,15) = 9.21, *p* = 0.0084*). The size of these main effects on PCoM is shown in Fig. 4C–F. As seen in Fig. 4F, a combination of Gain and Velocity resulted in a magnified influence on PCoM. Finally, a bootstrap test on the type of tracking method indicated that the device used did not exert a significant influence on PCoM (*p* = 0.088)^21^.

### The time of change of movement (tCoM)

**Figure 5:**
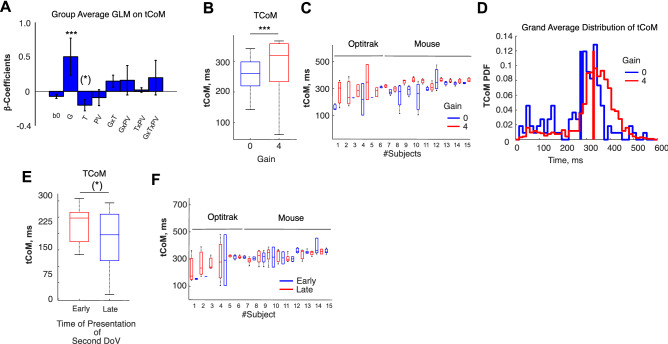
(**A**) Group average β-regression coefficients of GLMs performed on the time of Change of Movement (tCoM), as a function of: Gain (G), Time of presentation of the second distribution of visual reward (T), Peak tangential Velocity (PV), and their covariates. The tCoM exhibits a main increasing effect with G (F(15,1) = 7.30, *p* = 0.016*) and a decreasing trend with T (*p* = 0.071) (*****p < 1E−5, ****p < 1E−4, ***p < 1E−3, **p < 0.01, *p < 0.05). (**B**) Boxplot of group tCoM for Gain = 0 and Gain = 4. (**C**) Individual subject boxplots for Gain = 0 and Gain = 4. (**D**) Group average tCoM histogram as a function of G (Blue: G = 0; mean = 247.956 ms, σ = 51.62.ms/Red: G = 4; mean = 317.00 ms, σ = 116.55.ms). A post-hoc t-test yields a statistically significant difference between distributions at *p* = 2.98e−09. (**E**) Boxplot of the group tCoM as a function of DoVR presentation time. (**F**) Individual subject boxplots of tCoM as a function of DoVR presentation time.

We also calculated the time of CoM (tCoM) for every trial on which a CoM took place. The tCoM is defined as the time interval between the presentation onset of the second DoVR and the hard bend of the trajectory, indicating a change of movement towards the side of the rectangle opposite the initial choice. Consistent with the participant’s intent to maximize reward through precise arrival at the target, subjects displayed increased MTs and TTPDs as a function of increasing reward on baseline trials. We hypothesized that the reward to be gained by changing target side and the time of presentation of the second DoVR would influence the subjects’ urgency to adjust their motor trajectory and consequently their tCoM. To assess this, we performed a full GLM on each participants’ tCoM as a function of three factors: the gain (G) associated with the CoM, the time of presentation of the second DoVR, and the peak tangential velocity (V). Figure 5A shows the grand average GLM regression coefficients across participants. A subsequent F-test on each coefficient across subjects yielded a group effect of Gain on tCoM (F(1,15) = 7.30, *p* = 0.016*). In other words, the larger the Gain, the later the CoM (Fig. 5B–D). Interestingly, although velocity plays a role on the PCoM (Fig. 4E), it does not exert a significant effect on the tCoM (F(1,15) = 0.716, *p* = 0.79). Furthermore, there is a trend for the influence of the time of second DoVR appearance on the tCoM, although this effect does not survive multiple comparisons at the group level (F(1,15) = 4.14; *p* = 0.12), or a bootstrap test (*p* = 0.071)—Fig. 5E,F.

### Early versus late change of movement kinematics

To analyze the dynamics of the motor adjustments representative of CoM, we partitioned CoM movements into two phases: pre- and post-CoM, segmented at to the time post-movement onset at which both the tangential and radial velocity were minimal (Fig. 3B). This minimum indicated a hard bend of the trajectory toward the side of the target opposite their initial choice. Although the bend in trajectory signaled a switch, we hypothesized that the occurrence of a CoM depended on the state of the motor system. Thus, we expected the kinematic markers to be different prior to the presentation of the second DoVR on COM trials as compared to baseline trials.

**Figure 6:**
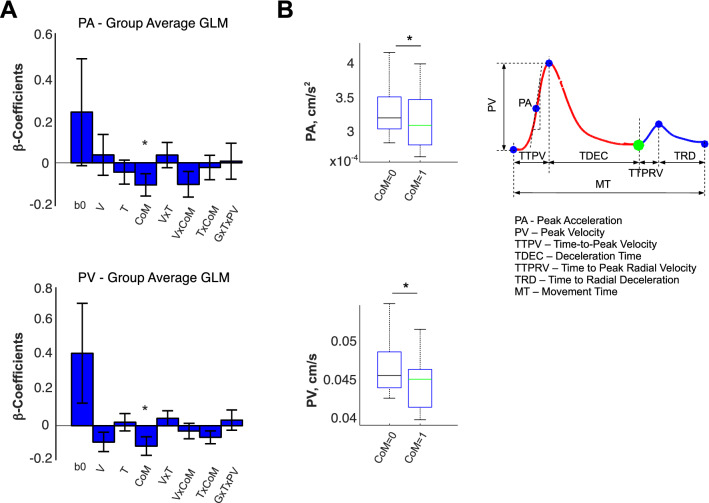
(**A**) Group average and standard deviation of the β-regression coefficients of GLMs performed on the Peak Velocity (PV) and the Peak Acceleration (PA), as a function of three factors: Visual reward (V), time of presentation of the second distribution of visual reward (T), a binary variable indicating whether there was a CoM, and their covariates; we included the offset coefficient (β_0_) for completion. Both PA (*p* = 0.031) and PV (*p* = 0.021) were sensitive to CoM, but not to V or T. (**B**) PV and PA as a function of CoM.

To test this hypothesis, we ran a full GLM on the kinematic markers during the pre-CoM interval (Peak Acceleration, Peak Velocity) as a function of the initially aimed at visual reward (V)—based on the first DoVR, its time (T) of presentation, a binary variable indicating a CoM, and its covariates (Fig. 6A,B). Figure 6A,B shows that both the PA and the PV were significantly smaller on trials where a change of trajectory occurred (PA: *p* = 0.021; PV: *p* = 0.031). Indeed, although the driving force underlying a CoM is a change in the location of the reward, our results would suggest that the initial state of the motor system, as characterized by the peak velocity and peak acceleration, is significantly different between CoM and non-CoM trials.

**Figure 7:**
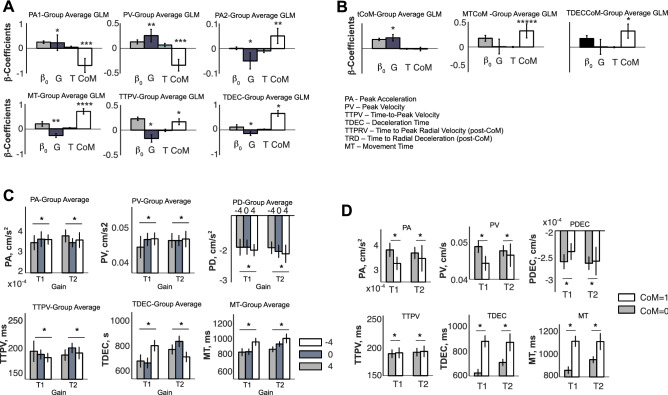
(**A**) Group average and standard deviation of the β-regression coefficients of GLMs performed on the Peak Acceleration (PA) and on the Peak Velocity (PV), Peak Deceleration (PD), Movement Time (MT), Time-To-Peak Velocity (TTPV), Time to Deceleration (TDEC) as a function of gain (G), time of presentation of the second distribution of visual reward (T), and a binary variable indicating CoM. Remarkably, all six kinematic factors showed a significant difference between COM trials and non-CoM trials. (**B**) Group average and standard deviation β-regression coefficients of GLMs performed on the time of CoM (tCoM), on the Movement Time after tCoM (MTCoM), and on the deceleration interval (TTPDCoM) of the second segment of the trajectory. (**C**) Group average and standard error across subjects for the PA, PV, PD, TTPV, TDEC, MT, as a function of Gain. (**D**) Group average and standard error across subjects for the PA, PV, PD, TTPV, TDEC, MT, as a function of whether or not a CoM occurred.

Finally, to provide a quantitative characterization of the relationship between the kinematic markers and CoMs, we performed a regression of kinematic markers, pre- and post-CoM (see “Methods” section), as a function of Gain (G), the presentation time of the second DoVR (T), and a binary variable CoM. Figure 7A,B shows the group regression coefficients for the GLMs pre- and post-CoM, respectively, whereas Fig. 7C,D shows the amplitude of these effects. F-tests across participant coefficients with Bonferroni correction for each GLM factor report two significant group effects. First, CoM exerts a significant positive effect on movement duration metrics: MT (F(1,15) = 20.55, *p* = 0.00039*), TTPV (F(1,15) = 7.05, *p* = 0.036*), MTCoM (F(1,15) = 37.54, *p* < 3.87E−5), and TDEC (F(1,15) = 18.71, *p* = 0.0012*), as well as on the peak deceleration: PD (F(1,15) = 7.36, *p* = 0.032*). Second, CoM exerts a negative effect on the amplitudes of early metrics, such as PA1 (F(1,15) = 8.28, *p* = 0.023*) and PV (F(1,15) = 12.38, *p* = 0.0062*)—Fig. 7A–C. In addition to this, Gain exerted an overall negative influence on movement duration metrics: TTPV (F(1,15) = 7.12, *p* = 0.035*) and overall MT (F(1,15) = 8.41, *p* = 0.022), as well as a positive influence on PD (F(1,15) = 6.41, *p* = 0.046*) and PV (F(1,15) = 8.05, *p* = 0.025*). The amplitudes of these effects are shown in Fig. 7C,D. In conclusion, these results strongly suggest that: although the trajectory bend occurs ~ 300 ms after the second DoVR presentation (Fig. 5), on trials where CoMs occur more frequently, movements tend to be longer and less energetic. Finally, the gain to be obtained by altering the ongoing movement is the major cause underlying CoMs.

## Discussion

The goal of this study was to investigate how changes in visual reward influence decision-making once commitment to an option has been made and the movement response is ongoing. Although previous evidence indicates that activity in the pre-motor cortex may encode several actions simultaneously^1,3,22^, reasonable doubt remains as to how that encoding extends to the execution phase, after a specific option has been selected and a movement is in progress. Here, we focused on decisions between motor trajectories where the option offering the greatest reward is most often selected, while equalizing all other factors. In this context, we hypothesized that a change in the distribution of reward at any point in time should dynamically adjust the desirability for each option, putatively resulting in a change of movement trajectory. To test this hypothesis, we performed an experiment in which human participants were instructed to freely select a reaching path trajectory from an origin to a wide rectangular target. The amount of reward attained was contingent upon the distribution of reward along the rectangle side as well as the end-point position upon arrival at the target. Reward distributions were altered, post-movement onset, on one third of all trials. Our results show that participants were most likely to alter their initially selected movement, even after their initial movement was ongoing, when the prospect of reward along a different path was better. Furthermore, changes of movement were more frequent when the initial movement parameters were slower, and required a duration, on average, inferior to the reaction time. Finally, the short latency of CoMs, together with the fact that the early time-to-peak-acceleration exhibited significant differences during CoM and non-CoM trials, strongly suggests that CoMs greatly depend on the current state of the motor apparatus.

### Baseline effect of visual reward

First, we examined the influence of visual reward on kinematic parameters during baseline trials. Several studies have shown that larger rewards tend to increase movement vigor^23,24^, energy^25^, and frequency^26^. In contrast, our results were consistent with a concern for precision, expressed by an increase in the overall movement time and duration of the deceleration phase (Fig. 2D). It is important to keep in mind that reward in this task was contingent on precision: responses hitting the extremes of the rectangle length were awarded a close to maximum reward, while those that missed the rectangle, received no reward. Thus, being conservative would be adaptive when there is a large reward at stake^27^.

### Gain and noise to change your motor plan

This study aimed at evaluating how online changes in reward distribution affect decision-making during the execution phase. Our results show that PCoM increased when the second DoVR resulted in a larger reward on the opposite side to the initial DoVR and when the initial movement was slow. Remarkably, PCoM in the absence of Gain occurs on 10–15% of all trials (Fig. 4A), implying that although most decisions aim at the largest reward prospect, subjects sometimes opt for lesser gain. Although this does not invalidate the main principle of seeking reward, it may be interpreted as an effect of neural noise, interfering with the commitment for a specific action in the presence of multiple options^3^.

In a similar fashion, tCoM exhibits the same increasing sensitivity to Gain as PCoM, a mild decrease with respect to the time of second DoVR presentation, and is insensitive to velocity. Consistent with the same hypothesis of neural noise, the mean tCoM is shorter when the Gain is zero than when the Gain is large (Fig. 5). This would imply that CoMs, whenever there is no reward to gain, are either made in the absence of proper processing of reward or guided by the concern of losing the reward offered by the alternative side^28,29^, and are biased by neural noise. This is also consistent with the fact that CoMs often occur close to the presentation of the second DoVR, rendering a hypothetical pre-frontal analysis of the second distribution unlikely. Moreover, this would be consistent with the fact that changing your movement when there is nothing to gain is counterproductive. Finally, tCoM occurs sooner when the presentation of the second DoVR occurs later (Fig. 5C), suggesting an increased urgency for change.

### Visual reward and ongoing behavior

Our analyses have also shown that the way CoMs occur during reaching movements is consistent with an interplay between two sequential movements. The first between the onset of movement and the offset of tangential velocity, and the second from the onset of radial velocity to the final offset. Importantly, the influence of the first DoVR on movement is constrained to a longer deceleration towards the target, consistent with a concern for precision and maximizing reward. By contrast, the second DoVR exerts a much broader influence on the specifics of movement kinematics, extending overall movement duration (MT, TTPV, TDEC, tCoM) and weakening movement intensity (PA, PV, PD), before and after the CoM (Figs. 6 and 7), shaping both the acceleration and deceleration phases and the way in which the movement is executed. Furthermore, our analyses of kinematics have also shown that, as early as the first peak acceleration, the movement exhibits significant differences between those trials where a CoM occurs vs those where there is no CoM. This implies that, although the hard bend in the trajectory occurs ~ 300 ms after the presentation of the second DoVR, the conditions necessary for the CoM to take place are already present around the initial PA (mean = 107 ms, std = 63 ms), shortly after the second DoVR has been presented. This is consistent with previous studies examining unpredictable target perturbations^30^, and together with the fact that PCoM is sensitive to movement velocity, strongly suggest that the state of the motor apparatus exerts a significant effect on PCoM. These very short latencies are also consistent with the extension of the affordance competition hypothesis to the execution phase^2,3,31^.

## Conclusion

The results from the analyses on PCoM and tCoM support the notion that visual reward exerts an influence not only on the choice itself but also on the way in which the movement is executed. These results also provide evidence for the role of goal-directed behavior when planning and executing motor decisions^2,32^, in line with previous studies. Participants changed their minds and adapted their trajectories based on reward, and these adjustments were enacted post-movement onset. Furthermore, reward exerted an influence on online feedback processes in several ways. First, there was a modulation of the time it takes to reprocess a new reward and change path trajectory (tCoM) as a function of the interplay between the currently selected path trajectory and reward, and the time of DoVR change. Thus, we change our mind if given sufficient time, and if the reward associated to a second option outweighs the current one. Second, in the context of voluntary movements, the motor system does not only take into consideration a variety of environmental factors and intrinsic biomechanical and external costs^3,7,33^, but also the perceived (cognitive) reward of the target, supporting the notion that decision-making models should factor in implementations of cost/benefit trade-offs. Third, the fact that changes of mind do occur on average quicker (~ 300 ms) than the RT prior to movement onset (~ 400 ms) supports the hypothesis that several movements may be planned in parallel, given that these adjustments must be made in a relatively small time-frame and require a rapid response. Fourth, feedback corrections appear to share the sophistication of the motor system for planning and executing motor actions; if the new distribution of visual reward shows that the alternative motor option offers a larger reward and there is enough time to adjust, then we are likely to change our mind.

### Limitations and future directions

This study focused on the influence of visual reward on decision-making between reaching movements during ongoing movements. Specifically, we focused on the subjective perception of reward that results from performing a movement as a function of a visual (non-monetary) reward. Under these conditions, our results yielded a tCoM twice as long as the RTs recorded for decisions where reward was absent and no monetary rewards were made, therefore suggesting the involvement of the reward system during deliberation. At a more methodological level, we acknowledge the potential influence of the reward distribution discontinuities at the target sides, on the choice of movement parameters. In a preliminary fashion, we controlled for this by analyzing the target arrival distributions, showing that the distributions were centered off the edge of the rectangle (Fig. 2B). This shows that the participants were concerned with gaining reward from the distribution presented, and that the concern for failure exerts a symmetrical influence on the arrival points. This symmetry is ensured by design, as the geometrical arrangement of the target with respect to the origin, have been designed to equalize motor costs and potential risks between target sides. This makes its influence on motor choices a minor concern.

## Methods

### Participants

Fifteen right-handed individuals (5 M and 10F; Mage = 24.4 years, SD = 5.8), participated in this research study. Nine other participants served as pilots to test the experimental setup, the recording process, and the custom scripts controlling the task flow; their data was not considered in further analyses. All participants had normal or corrected-to-normal vision and hearing and did not suffer from any known neurological disorders. Informed consent was obtained following the guidelines established by the local ethics committee and all participants received monetary compensation (10€/h) for their participation, regardless of completion. The ethics protocol for this study was approved by the Clinical Research Ethics Committee (CEIC-Parc Salut MAR) of the Pompeu Fabra University-Hospital del Mar, with Reference #2015/6085/I. All methods were carried out in accordance with relevant guidelines and regulations.

### Experimental setup and task apparatus

Participants were seated in a comfortable chair, facing the experimental table, with their chest approximately 10 cm from the table edge and both lower arms resting on its surface. The table defined the plane where reaching movements were to be performed. On the same table, approximately 60 cm away from the participant’s sitting position, we placed a vertically-oriented, 24″ Acer G245HQ computer screen (1920 × 1080). This monitor was connected to an Intel i5 (3.20 GHz, 64-bit OS, 4 GB RAM) computer that ran custom-made scripts which controlled task flow, programmed using OpenFrameworks v.0.9.8 software. The screen was used to show the geometrical arrangements and related stimuli on each trial. A small cross (1 × 1 cm), whose position was synchronized with the planar coordinates of the end-point as it slid along the horizontal plane (table), was used to show the participant’s corresponding movement in the vertical plane on the screen.

As part of the experiment, subjects had to respond by performing overt movements with their arm along the table plane. Due to operational laboratory constraints, their movements were recorded with two end-point tracking methods: (1) for participants 1–5; with an Optitrak motion tracking system (Optitrak, Inc), which tracked the position of a spherical marker placed on top of the nail of the right-hand index finger, as it slid across the table; (2) for participants 6–15; with a light computer mouse (Logitech, Inc) that tracked hand position. The weight of the mouse (~ 0.1 kg) was less than 1% of the total weight of the arm (~ 5 kg). A comparison of both methods indicated that the trajectories and velocity profiles recorded with both methods were virtually identical (see Supplemental Figs. S1 and S2). Subjects were instructed to apply minimal pressure but maintain end-point contact with the table surface at all times. They were permitted to minimally lift their elbow off the table to diminish this effect. Given that the monitor was placed vertically and movements were performed horizontally (along the table surface), the movement component along the sagittal plane was rotated 90°, to the frontal plane, to show displacements of the end-point on the screen. The transverse movement component was shown unaltered. We used a chinrest to stabilize posture and fix the head position at a predetermined distance from the screen. Data analyses were performed with custom-built MATLAB scripts (The Mathworks, Natick, MA), licensed to the Pompeu Fabra University.

### Experimental task

We designed a reaction time task, consisting of 630 trials, performed in a single session. The participant was asked to perform a reaching movement from a circular origin cue (diameter: 1.5 cm) to a wide rectangular target (width: 10 cm; depth: 1 cm), placed 15 cm apart and rotated 135 counter-clockwise (Fig. 1), with the goal of maximizing reward. We explained to the subject that reward in this task was dependent upon the arrival position relative to the long rectangle side, and upon the distribution of visual reward. The orientation of the target was selected in order to equalize motor costs for movements towards the right and left side of the rectangle, as it coincides with the direction of minimal arm mobility^34^. In other words, movements towards either side of the rectangle implied the same motor cost^31^. Furthermore, since the goal of the task was to assess the influence of reward, at the beginning of all trials, we showed one of three bimodal distributions of visual reward (DoVR): 3–3, 1–5, 5–1. These DoVRs peaked at the right/left edges of the rectangle’s long side and decreased towards zero when approaching its center (Fig. 1C). They were also equal to zero off the right/left sides of the rectangle, meaning that reaching movements that missed the target would not yield any reward. The subject was instructed to make a reaching movement from the origin cue to a freely selected position along the long side of the rectangular target while attempting to maximize reward. The reward obtained on each trial was dependent on arrival position along the length of the rectangle as well as the final DoVR.

The task contained two types of trials: baseline and change of movement (CoM) trials, which were randomly interspersed. Each participant performed 7 blocks of 90 trials in a single session (~ 1 h 15 min). Each block consisted of 72 baseline trials (24 repetitions of each DoVR) and 18 CoM trials. There were 18 types of CoM trials, as a function of the change of DoVR (3–3/1–5; 3–3/5–1; 1–5/3–3; 1–5/5–1; 5–1/3–3; 5–1/1–5) and the time at which the DoVR occurred, measured from the onset of movement (Early (E; t < 80 ms), Medium (M; 80 ms < t < 140 ms) or Late (L; t > 140 ms ± 30 ms)). Each block contained one trial of each possible CoM type. Trial order was counterbalanced and randomized both within and across blocks. The second DoVR was shown for a duration of 200 ms.

Real-time, visual feedback of hand position was provided during the trial by a 1 cm cross shown on the screen, synchronized with the position of the participant’s right hand on the experimental table. The time-course of each trial type can be seen in Fig. 1A,B. A baseline trial began when the origin was shown on the screen and the subject entered the cue. Approximately 1 s later, the rectangular target and the initial DoVR were shown (Fig. 1A,B). After a 1 s observation interval, a GO signal was given by making the origin cue disappear. The participant was instructed to perform their selected path trajectory towards the position along the side of the rectangle they deemed most rewarding. If the subject left the origin before the GO signal was given, the trial was invalidated, the experimental arrangement disappeared, and the participant was shown a blank screen where they had to wait until the regular trial duration of 7 s elapsed. Correct target entry resulted in the rectangle turning green. After 500 ms of holding position at the target, the reward associated with the specific end position was shown for a duration of 500 ms. The screen turned white for an interval that equalized the entire trial duration to a fixed overall duration of 7 s. The CoM trials followed the same time-course as the baseline trials, except for the appearance of a second DoVR, which changed 80–200 ms post-movement onset. At the beginning of each block, subjects were verbally reminded that their goal remained to maximize reward and that, on CoM trials, they may have to change their movement to attain that goal. Feedback was provided at the end of each trial in the form of a red horizontal bar, shown for 1000 ms. The length of the bar was proportional to the reward related to their reaching movement. The inter-trial interval was modulated to maintain a fixed 7 s, to prevent participants from increasing their speed to maximize reward. For the following analyses, we discarded trials with a response time greater than 7 s, as well as trials where the subject entered the target rectangle through the sides or top, and/or where the participant left the origin before the GO signal.

### Statistical analyses

#### The probability of a change of movement (PCoM)

We calculated the probability of a change of movement (PCoM; Fig. 4), by counting the times each subject changed their movement on CoM trials, and fitting a binomial distribution for the proportion of changes over the total amount of trials with the binofit function provided by MATLAB. A binomial distribution is characterized by a single p-parameter, which in our case captures the PCoM.

To specifically analyze changes of movement, we operationally defined: the notion of prospect gain (G) as the difference in reward between the currently aimed at rectangle side and its alternative, after the second DoVR onset. In other words, G quantifies the reward to be gained if the trajectory were to be re-directed to the opposite side of the target, vs the reward to be obtained if the subject sticks to the original choice. Furthermore, we also defined a binary variable (Tracking—TR), which indicated the movement tracking device for each participant (0-Optitrak, 1-Mouse). We then calculated the binomial p-parameter for each participant for each possible combination of experimental factors: as a function of G (− 4, 0, 4), for early/late presentation times of the second DoVR (T), and for slow/fast movements, according to the Peak Velocity (PV) of the behavioral response. Both T and PV were classified as Early/Late and Slow/Fast using median splits within the T and PV distributions of each individual participant. We then used a mixed-effects model, and fitted a full GLM of the resulting p-binomial parameter against three factors: G, T, PV, and their covariates for each individual subject. We also incorporated the Tracking (TR) binary variable to make a global fit on the entire dataset and to measure the influence of TR on PCoM. Group significance was established by Bonferroni corrected F-/t-tests on each of the regression coefficients (Fig. 4B; signaled by a^*^), and a permutation test of the influence of the tracking device on group PCoM, with *p* < 0.05. For presentation purposes, we fitted a parametric sigmoidal curve to the *p*-parameter obtained as a function n of prospect gain (G) for each individual subject—Eq. (1).

1 $$P_{CoM} = \frac{{e^{{b_{1} G + b_{2} }} }}{{1 + e^{{b_{1} G + b_{2} }} }}$$

#### The time of change of movement (tCoM)

To test our hypothesis, the analysis of the temporal dynamics of the CoM was crucial. Thus, we placed specific emphasis on the analysis of the onset of the second DoVR and that of the change of motor trajectory. To this end, we calculated the time intervals before the onset of the second DoVR and the hard bend in the trajectory, which coincided with the moment at which tangential and radial velocities were minimal. We tested whether the time of change of movement (tCoM) was dependent on three factors: reward gain (G), the time at which the second DoVR was presented (T), and the Peak Velocity of the ongoing movement (PV)—which we used as proxies of the motor apparatus state during movement. As for the PCoM we used a mixed-effects model approach by regressing a full-GLM for the three aforementioned variables and their covariates, as well as the TR variable indicating the tracking device for that subject. We performed an individual fit per subject within a single global regression, which yielded a set of regression coefficients per subject and a global regression coefficient for the TR variable (0-Optitrak, 1-Mouse). We calculated the average and standard error on beta regression coefficients for G, T, PV and their covariates (Fig. 5A). Statistical significance was assessed via Bonferroni corrected F-/t-tests for G, T and PV, and covariates (signaled by a^*^). A permutation test was performed to assess the influence of the TR variable on tCoM. We also calculated the histograms of tCoMs for each G = 0 and G = 4 across subjects (Fig. 5D), and plotted the influence of significant effects (Gain and Time) on tCoM, at the group level (Fig. 5B,D and E) and for each individual participant (Fig. 5C,F).

#### Kinematic markers

In addition to PCoM and tCoM, which are fundamental metrics underlying changes in movement, we considered it of interest to explore other metrics underlying CoM such as those found within the movement trajectories, velocities, and accelerations. We used custom-made MATLAB scripts to this end. To characterize the dynamics of CoM, we also analyzed the sequence of kinematic metrics typical of a reaching movement (Figs. 6 and 7). For each trial, we calculated the following kinematic markers from the tangential velocity: peak acceleration (PA), time-to-peak acceleration (TTPA), peak velocity (PV), time-to-peak velocity (TTPV), peak deceleration (PD), time-to-deceleration (TDEC), and overall movement time (MT). On CoM trials, we also calculated kinematic markers as a function of the radial velocity after the CoM including: peak radial velocity (PRV), the time-to-peak radial velocity (TTPRV), the overall switch movement time from the trajectory hard bend to the movement offset (MTCoM), the time of deceleration, from the TTPRV to the movement offset—Fig. 3C.

#### Analysis of kinematics

A complementary analysis to the metrics of PCoM and tCoM, we examined the extent to which potential changes of mind influenced movement-related parameters. With this in mind, we distributed the kinematic markers as pre- and post-CoM as a function of whether they preceded or succeeded the CoM—Fig. 3C. Note that this distinction was possible because, at the switch on CoM trials the participants’ tangential and radial velocities were very close to zero. Consequently, we performed two groups of full GLMs across subjects. First, on the pre-CoM factors: peak acceleration (PA), Time-To-Peak-Acceleration (TTPA), Peak Velocity (PV), and Time-to-Peak-Velocity (TTPV) as a function of three factors: Gain (G), the time of presentation of the second DoVR (T), a binary variable indicating the presence of a CoM, and their co-variates. Statistical group significance was established via Bonferroni corrected F-/t-tests on the regression coefficients obtained across participants for each individual factor (signaled with an * on each p-value)—Fig. 6. Second, on the post-CoM kinematic markers, namely the movement time after CoM (MTCoM) and deceleration time post-CoM (TDECCoM). We performed a GLM as a function of G, T, and CoM factors (Fig. 7). Furthermore, Bonferroni corrected F-/t-tests were performed on the regression coefficients to establish statistical significance.

## Supplementary information

Supplementary Figures
