# Supplementary material for: Visual-reward driven changes of movement during action execution
Source: Sci Rep. 2020 Sep 23;10:15527. doi: 10.1038/s41598-020-72220-2 (PMC7511350; doi:10.1038/s41598-020-72220-2)
Supplement: Supplementary file 1 — Supplementary Figures [file 41598_2020_72220_MOESM1_ESM.pdf]

Angela B. Martí-Marca, Gustavo Deco and Ignasi Cos

Supplementary Material for the Manuscript: Visual-Reward Driven Changes of Movement during Action Execution

We added two figures as additional material. Supplemental Figure 1 shows the trajectories and velocities of four typical participants, for each distribution of visual reward (3-3, 5-1, 1-5) during the baseline condition. For comparative purposes, the first two were recorded with the Optitrak and the second two with the Mouse. Supplemental Figure 2 shows a scatterplot of the MT vs PV means for each individual subject, and the average and standard error of the PV and MT as a function of the tracking method. T-tests between them did not yield significant differences ($P>0.05$) in all cases.

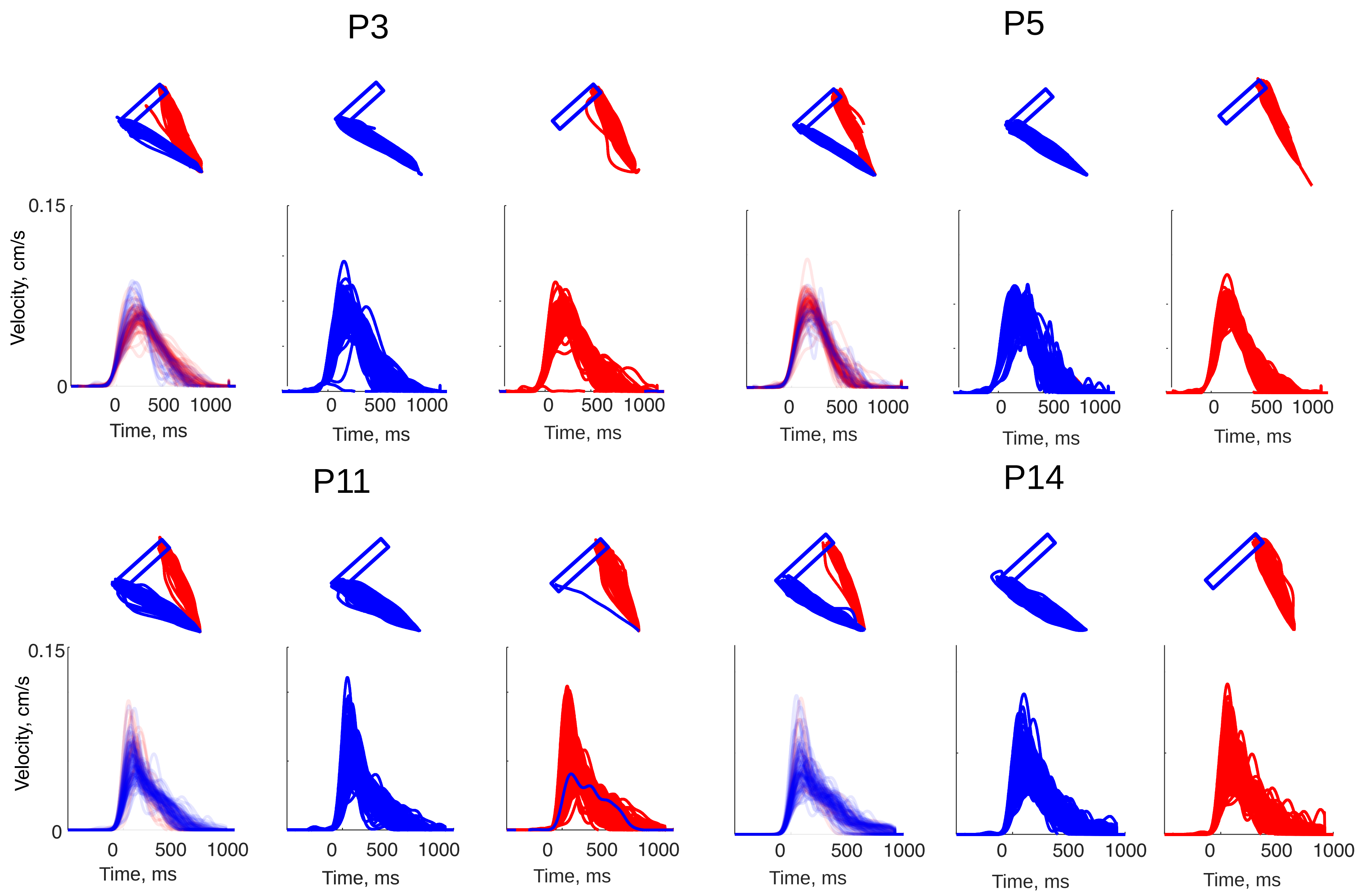

Supplemental Figure 1. Baseline sample data. End-point trajectories and Tangential Velocities for P3 & P5 (Optitrak recorded), P11 & P14 (Mouse recorded), aligned on movement onset, during baseline trials, for each distribution of value 3-3, 5-1, 1-5, from left to right, respetively.

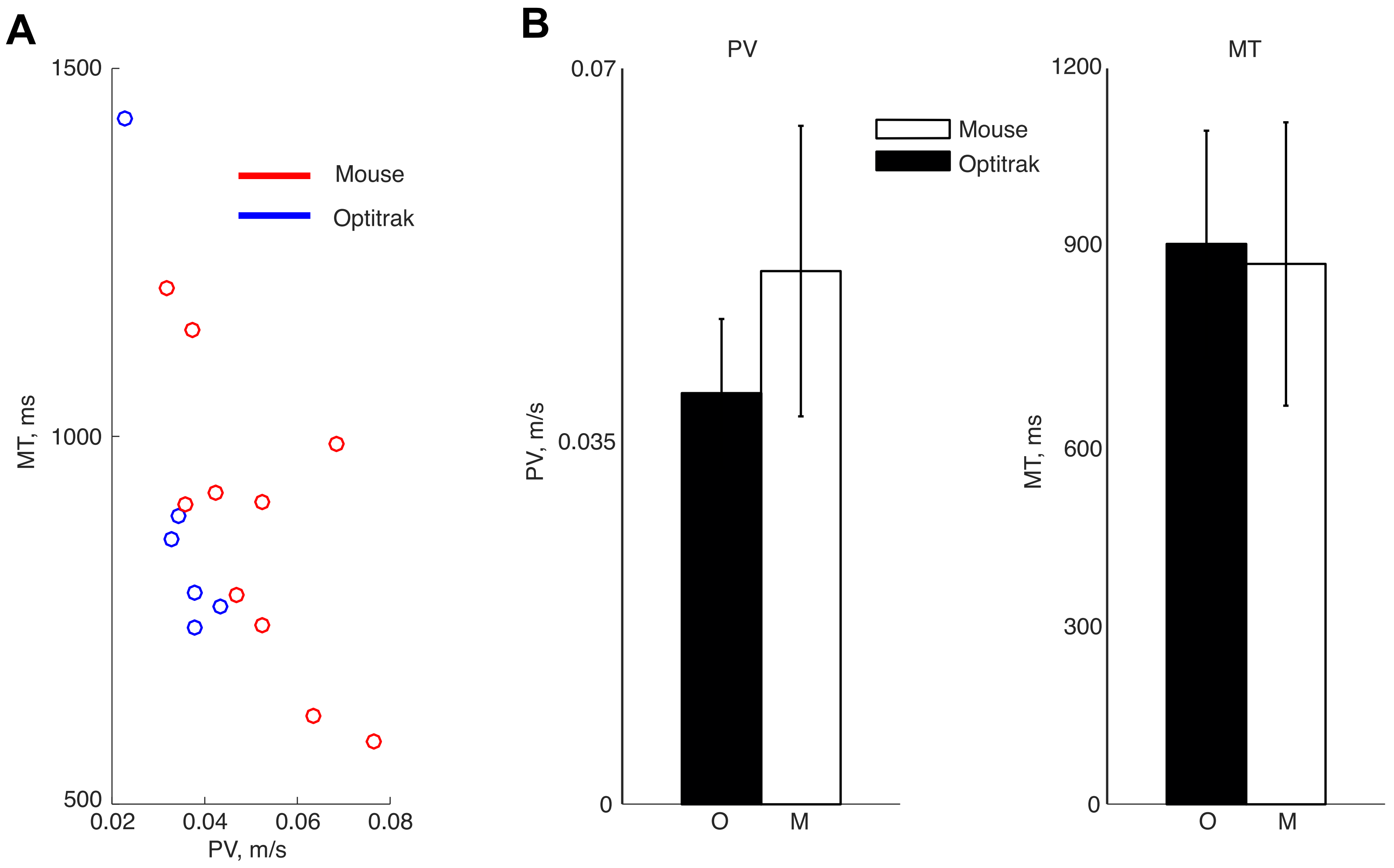

Supplemental Figure 2. **A**. Average Movement Time vs Peak Velocity for each individual subject. Blue – Optitrak recorded; Red – Mouse recorded. **B.** Mean and Standard Deviation of the Group PV and MT grouping subjects as a function of whether their trajectories were tracked by the Optitrak or by the Computer Mouse.
